# 2-Acet­amido-4,5-di­nitro­toluene: a test mol­ecule for the CCDC ‘Blind Structure Prediction Test, 2004’. Corrigendum

**DOI:** 10.1107/S1600536812039670

**Published:** 2012-09-26

**Authors:** David J. Watkin, W. D. S. Motherwell, Richard I. Cooper, Stefan Pantos

**Affiliations:** a Chemical Crystallography, Chemistry Research Laboratory, University of Oxford, Mansfield Road, Oxford OX1 3TA, England; b Cambridge Crystallographic Data Centre, 12 Union Rd, Cambridge CB2 1EZ, England

## Abstract

The name of one of the authors in the paper by Watkin *et al.* [*Acta Cryst.* (2004), E**60**, o2295–o2297] is corrected.

## Abstract 2

Corrigendum to *Acta Cryst.* (2004), E**60**, o2295–o2297.

 In the paper by Watkin *et al.* (2004) ▶, the name of the third author is given incorrectly. The correct name is given above.
